# Advanced-Curative Oral-Health Entitlement, Basic Restorative Service Availability, and Untreated Dental Caries in Primary Dentition: A Cross-National Ecological Study

**DOI:** 10.3390/healthcare14213609

**Published:** 2026-10-25

**Authors:** Semih Ercan Akgün, İrem Okumuş

**Affiliations:** 1 Department of Dentistry Services, Vocational School of Health Services, Ondokuz Mayıs University, 55200 Samsun, Türkiye; semihercan.akgun@omu.edu.tr; 2 Department of Pediatric Dentistry, Faculty of Dentistry, Recep Tayyip Erdogan University, 53020 Rize, Türkiye

**Keywords:** dental caries, tooth, deciduous, health services accessibility, universal health coverage, ecological study

## Abstract

**Highlights:**

**What are the main findings?**

• In the primary 92-country model, advanced-curative entitlement modified the country-level association between reported restorative service availability and untreated caries prevalence (interaction β = 9.00 percentage points; 95% CI, 1.71–16.29; *p* = 0.016).
• The No/Available category had a lower adjusted predicted prevalence than Yes/Available (difference, −6.29 percentage points; 95% CI, −11.06 to −1.52; Holm-adjusted *p* = 0.031), without implying that absent entitlement is beneficial.

**What are the implications of the main findings?**

• Entitlement and reported service availability capture distinct dimensions of national oral-health coverage.
• Monitoring should also examine affordability, geographic access, service use, and care delivery; the ecological findings cannot establish policy effects.

**Abstract:**

**Background/Objectives:** Formal oral-health entitlement does not necessarily imply available services or effective access. We examined whether the country-level association of basic restorative service availability with untreated caries in primary teeth among children aged 1–9 years differed by advanced-curative entitlement. **Methods:** Data for 2021 were harmonized from IHME, WHO, FAOSTAT, and the World Bank for 92 countries with complete data. Ordinary least squares regression with HC3-robust covariance included entitlement, reported restorative service availability, their prespecified interaction, log GDP per capita, sugar availability, and WHO region. **Results:** Mean untreated caries prevalence was 45.10% (SD, 7.60). The interaction was positive (β = 9.00 percentage points [pp]; 95% CI, 1.71–16.29; *p* = 0.016). Among countries without advanced-curative entitlement, availability was associated with lower prevalence (β = −6.62 pp; 95% CI, −12.23 to −1.02; *p* = 0.021). Compared with Yes/Available, the No/Available category had a 6.29 pp lower adjusted predicted prevalence (95% CI, −11.06 to −1.52; Holm-adjusted *p* = 0.031). The child-population-weighted interaction was not statistically significant (β = 6.11 pp; 95% CI, −1.01 to 13.24; *p* = 0.092). **Conclusions:** Entitlement and reported availability are clinically non-equivalent indicators and cannot establish policy–service alignment or effective access. Findings are ecological associations, not evidence of policy effects.

## 1. Introduction

Untreated caries in primary teeth remains a substantial child-health burden, with geographic and socioeconomic inequalities [1]. Systematic reviews show that early childhood caries (ECC) is common among preschool children, although prevalence varies widely by setting [2,3]. ECC is associated with poorer oral health-related quality of life [4], and poor oral health has been linked to school attendance and performance [5]. Dietary sugars are an established caries risk factor [6]; however, population-level variation also reflects socioeconomic inequalities, access to care, financing, and the organization of oral-health services [1,3,7,8].

Integrating oral health into universal health coverage (UHC) requires attention to both benefit-package entitlement and service availability [9,10]. These indicators describe distinct constructs. Here, advanced-curative entitlement denotes inclusion of more complex dental care in the largest government health-financing scheme, whereas basic restorative service availability denotes reported national availability of procedures for treating existing decay. Both refer to 2021, but they represent different levels of dental care and cannot be treated as clinically equivalent or as direct measures of policy–service alignment.

Formal entitlement alone does not demonstrate affordability, geographic accessibility, treatment utilization, or effective care delivery [10,11]. Comparative research has shown that publicly funded child dental coverage can coexist with access barriers and substantial out-of-pocket costs [12]. Accordingly, the two WHO indicators were analyzed jointly to investigate associations, not to evaluate implementation success.

Previous international research has examined several components of the relationship between health-system characteristics and childhood oral-health outcomes, but through different policy and service constructs. Folayan et al. evaluated ECC prevalence across countries in relation to the UHC service coverage index and national health expenditure; in their adjusted analysis, health expenditure was associated with ECC prevalence, whereas the UHC service coverage index was not independently associated with the outcome [13]. Foote et al. examined 19 OECD countries and reported associations between preventive dental policy, oral-health expenditure, and DMFT among children and adolescents aged 12–18 years [14]. Henschke et al. compared public oral-health coverage and oral-health outcomes across five European countries and demonstrated substantial differences in both coverage arrangements and age-specific oral-health outcomes [15]. At a broader international level, Gaffar et al. identified marked variation between countries in national oral-health policies and in the scope of oral-health coverage for young children [16]. More recently, a European ecological analysis linked statutory dental coverage and dental financing with untreated caries and unmet dental needs, providing further evidence that health-system characteristics may be associated with population oral-health outcomes [17]. Together, these studies have examined broad UHC, health expenditure, preventive regulation, statutory coverage, and national oral-health policies. However, it remains unclear whether the country-level association between advanced-curative benefit-package entitlement and untreated caries in the primary dentition differs according to reported basic restorative service availability. In particular, evidence remains limited on the joint assessment of these two dimensions using a common reference year and a broad multinational sample.

To address this gap, the present cross-national ecological study examined whether the association between reported basic restorative service availability and country-level untreated caries prevalence among children aged 1–9 years differed according to advanced-curative entitlement status. We prespecified an entitlement-by-availability interaction and adjusted for GDP per capita, sugar availability, and WHO region. We hypothesized that the association between restorative service availability and untreated caries prevalence would differ according to advanced-curative entitlement status.

## 2. Materials and Methods

### 2.1. Study Design and Data Sources

This cross-national, cross-sectional ecological study used countries as the analytical units to assess oral-health-system indicators in relation to untreated caries prevalence among children aged 1–9 years. Individual-level and causal inferences were not intended.

The reference year for the primary outcome and the principal policy, service, economic, and dietary indicators was 2021. Country-level estimates of untreated caries in deciduous teeth for 2021 were obtained from the Institute for Health Metrics and Evaluation (IHME; Seattle, WA, USA) Global Burden of Disease (GBD) 2023 release. The designation “2023” refers to the database release rather than the year of the prevalence estimate. Oral-health entitlement indicators were obtained from the World Health Organization (WHO) Global Health Observatory, based on the 2021 Health Technology Assessment and Health Benefit Package Survey. These included advanced-curative, essential-curative, and routine/preventive oral-health care. These indicators describe benefit-package inclusion reported for the 2021 reference year; however, the analyzed data did not provide information on when these policies were introduced or how long they had been implemented.

Reported restorative service availability was obtained from the 2021 WHO NCD Country Capacity Survey using the indicator “Basic restorative dental procedures to treat existing dental decay.” National sugar availability was derived from FAOSTAT Food Balances using “Sugar (Raw Equivalent), Food supply quantity (kg/capita/year)” for 2021. Gross domestic product per capita was obtained from the World Bank (Washington, DC, USA) World Development Indicators using “GDP per capita (current US$; NY.GDP.PCAP.CD)” for 2021. Dentist density, expressed as dentists per 10,000 population, was obtained from the WHO Global Health Observatory using the 2021 observation where available or the nearest observation within 2019–2023 otherwise. Dentist density was summarized descriptively and incorporated as an additional covariate in sensitivity analyses but was not included in the primary adjustment set. Data sources, reference years, data availability, and analytical roles are summarized in Supplementary Table S1. This study was reported in accordance with applicable recommendations of the Strengthening the Reporting of Observational Studies in Epidemiology (STROBE) statement. Because the study used exclusively publicly available, aggregated, non-identifiable country-level data, formal ethics committee approval was not required.

### 2.2. Study Population and Data Harmonization

The initial sampling frame consisted of all country-level locations for which a 2021 estimate of caries of deciduous teeth was available in the selected IHME dataset. Regional aggregates, global estimates, income-group aggregates, and other supranational observations were excluded.

ISO3 and a UN M49–ISO. Records were linked primarily through ISO 3166-1 alpha-3 (ISO3) country codes [18]. WHO provided ISO3 codes; World Bank and FAOSTAT identifiers were standardized using ISO3 and a UN M49–ISO crosswalk, respectively. IHME locations were matched to national identifiers and verified country names, including review of naming variants and territories.

Source files were preserved. Before merging, identifiers, years, duplicates, missingness, and implausible values were examined; conflicting duplicates were checked against source records rather than averaged. Supplementary Table S2 provides the harmonized dataset and country inclusion status.

The primary analysis required complete outcome, advanced-curative entitlement, restorative availability, GDP, sugar availability, and WHO-region information. Missing entitlement or availability was not treated as a negative response. Complete-case analysis avoided imputing unobserved categorical national policy characteristics, but may introduce selection bias. Missingness and included-versus-excluded comparisons are reported in Supplementary Table S3, while sequential sample selection and variable-level missingness are detailed in Supplementary Table S4.

### 2.3. Outcome and Oral-Health-System Exposures

The primary outcome was the 2021 prevalence of untreated caries of deciduous teeth among children aged 1–9 years, expressed as a percentage.

IHME provided 2021 prevalence estimates for three mutually exclusive age groups, namely, 12–23 months, 2–4 years, and 5–9 years, reported as estimated prevalent case counts (Number) and prevalence proportions (Percent). These estimates were combined by dividing the sum of age-specific prevalent case counts by the sum of their implied population denominators rather than calculating the arithmetic mean of age-specific prevalence percentages. The resulting country-level prevalence was population-weighted according to the age distribution within each country and was not age-standardized. Consequently, differences in the relative sizes of the age groups may influence comparisons between countries. Although the source GBD estimates included uncertainty bounds, joint posterior draws and correlations between age groups were not available in the extracted dataset. Therefore, uncertainty from the underlying GBD estimates was not propagated to the aggregated outcome, and the regression confidence intervals were calculated conditional on the GBD point estimates.

For each age group, the implied population denominator was calculated as:

N_a_ = C_a_/P_a_

where C_a_ represents the estimated number of prevalent cases and P_a_ represents prevalence as a proportion. The country-specific prevalence for children aged 1–9 years was then calculated as:

P_1–9_ = [(C_12–23_m + C_2–4_y + C_5–9_y)/(N_12–23_m + N_2–4_y + N_5–9_y)] × 100

The outcome was analyzed continuously. Arithmetic consistency of the aggregation was checked, but an independently reported or age-standardized 1–9-year estimate was unavailable for external validation.

Advanced-curative entitlement was classified Yes/No according to the inclusion of advanced-curative oral/dental care in the largest government health-financing scheme in the 2021 WHO benefit-package survey. It includes selected complex restorations and root-canal treatment [19]. A ‘No’ response does not exclude coverage of basic restorative care or alternative public/private financing.

The second exposure, basic restorative service availability, was derived from the 2021 WHO NCD Country Capacity Survey indicator “Basic restorative dental procedures to treat existing dental decay.” Countries were classified as Available when these procedures were reported as “generally available” (reaching at least 50% of patients in need) and as Unavailable when reported as “generally not available” (reaching fewer than 50% of patients in need) [20]. Thus, Unavailable does not necessarily indicate a complete absence of restorative services. This country-level indicator represents reported service availability rather than geographic accessibility, individual treatment utilization, quality of care, or treatment receipt among children. Furthermore, the basic restorative procedures assessed by this indicator are not identical to the advanced-curative interventions represented by the entitlement indicator.

The indicators defined four descriptive joint categories: Yes/Available, Yes/Unavailable, No/Available, and No/Unavailable (advanced-curative entitlement/restorative availability). These are not validated policy–service alignment or implementation-gap measures. Yes/Available was the prespecified reference for marginal contrasts; No entitlement and Unavailable services were the main-effect reference levels in the interaction model.

Essential-curative and routine/preventive entitlement were examined separately as secondary exposures, to avoid overlap among benefit-package indicators.

### 2.4. Covariates

The prespecified adjustment set comprised GDP per capita, sugar availability, and WHO region. These covariates were selected based on conceptual relevance and cross-national data availability to provide partial contextual adjustment rather than comprehensive control of confounding. GDP per capita was included as an indicator of national economic development and resource availability, which may influence both the organization and financing of oral-health services and population-level oral-health outcomes. The 2021 GDP per capita values were natural-log-transformed to account for their right-skewed distribution. Sugar availability was included as a country-level indicator of the dietary risk environment, given the established association between sugar consumption and dental caries. However, national food-supply availability does not directly represent individual sugar intake, consumption frequency, or dietary exposure among children. WHO region was included as a categorical covariate to account partially for broader geographical, epidemiological, and health-system differences between countries. Sugar availability was expressed as grams per person per day, with FAOSTAT values reported in kilograms per capita per year converted according to:

Sugar (g/day) = [kg/capita/year × 1000]/365

Sugar availability was modeled per 10 g/day increment. Countries were grouped into the six WHO regions: Africa, Americas, Eastern Mediterranean, Europe, South-East Asia, and Western Pacific.

Dentist density was excluded from the primary adjustment set because it may partly reflect service-system capacity and because observations differed in completeness and year. Sensitivity models included the nearest 2019–2023 value and, separately, exact 2021 values. These smaller samples were not directly comparable with the primary sample, and workforce adjustment was not assumed to remove all confounding.

### 2.5. Statistical Analysis

Analyses were performed in R version 4.6.1 (R Foundation for Statistical Computing, Vienna, Austria). Continuous variables were summarized as means (SD) or medians (IQR), and categorical variables as *n* (%). Across joint categories, Welch ANOVA compared caries prevalence and sugar availability; Games–Howell tests assessed post hoc prevalence differences; Kruskal–Wallis tests with ε^2^ evaluated GDP and dentist density; and Pearson’s χ^2^ with 20,000 label permutations and Cramér’s V assessed WHO-region distributions. These comparisons were descriptive.

The primary model was unweighted country-level ordinary least squares regression (N = 92) with HC3 heteroskedasticity-robust covariance. Predictors were advanced-curative entitlement, restorative service availability, their prespecified interaction, log-transformed GDP per capita, sugar availability (per 10 g/person/day), and WHO region. The interaction, retained regardless of significance, tested whether the availability–prevalence association differed by entitlement status. Coefficients were expressed as percentage-point differences with 95% confidence intervals (CIs) and two-sided *p* values. Conditional availability associations within entitlement strata were derived from linear combinations of model coefficients and their HC3 covariance matrix; these comparisons used unadjusted *p* values and were interpreted descriptively. Adjusted prevalence was marginally standardized over the observed covariate distribution. Three prespecified contrasts versus Yes/Available were evaluated with 95% CIs and Holm-adjusted *p* values.

Separate secondary models evaluated routine/preventive and essential-curative entitlement, each adjusted for the primary covariates using all available complete cases; these entitlement variables were not entered together. Prespecified sensitivity analyses included beta regression (logit link and HC3 covariance), adjustment for dentist density (nearest 2019–2023 observation or exact 2021 observation), an alternative essential-curative entitlement × restorative service availability interaction, exclusion of countries with Cook’s D > 4/N, and child-population-weighted least squares. Weights were the summed implied age-specific population denominators for children aged 1–9 years from IHME GBD 2023. The unweighted model remained primary because countries were the analytical units and contributed equally; population weighting represented an alternative weighting scheme. Dentist-density models used smaller complete-case samples.

Exploratory, non-prespecified analyses repeated the primary HC3 model for ages 2–4 and 5–9 years in the same 92 countries and added the proportion aged 5–9 years within each country’s implied 1–9-year population to the primary 1–9-year model. *p* values from these exploratory analyses were not adjusted for multiple comparisons.

To evaluate potential selection bias, variable-specific missingness was assessed across the initial 204 national-level locations. Countries were assigned to the first applicable exclusion stage: missing advanced-curative entitlement, restorative availability, or sugar availability; assignment did not imply completeness of remaining variables. Selection-stage and included-country distributions were examined by WHO region and quartiles of observed 2021 GDP per capita (not official World Bank income groups). Included versus excluded countries were compared using Welch’s t tests (caries prevalence and sugar availability), the Mann–Whitney U test (GDP), and Pearson’s χ^2^ with 20,000 label permutations (WHO region); inclusion across GDP quartiles used Pearson’s χ^2^. These comparisons were exploratory and descriptive, not tests of representativeness or missing-data mechanisms. All tests were two-sided (α = 0.05), with emphasis on estimates and 95% CIs.

## 3. Results

### 3.1. Study Sample and Descriptive Categories

In the 92-country primary sample, mean untreated caries prevalence among children aged 1–9 years was 45.10% (SD, 7.60). Advanced-curative entitlement was reported in 63 countries (68.5%), and basic restorative services were available in 65 (70.7%).

**Figure 1:**
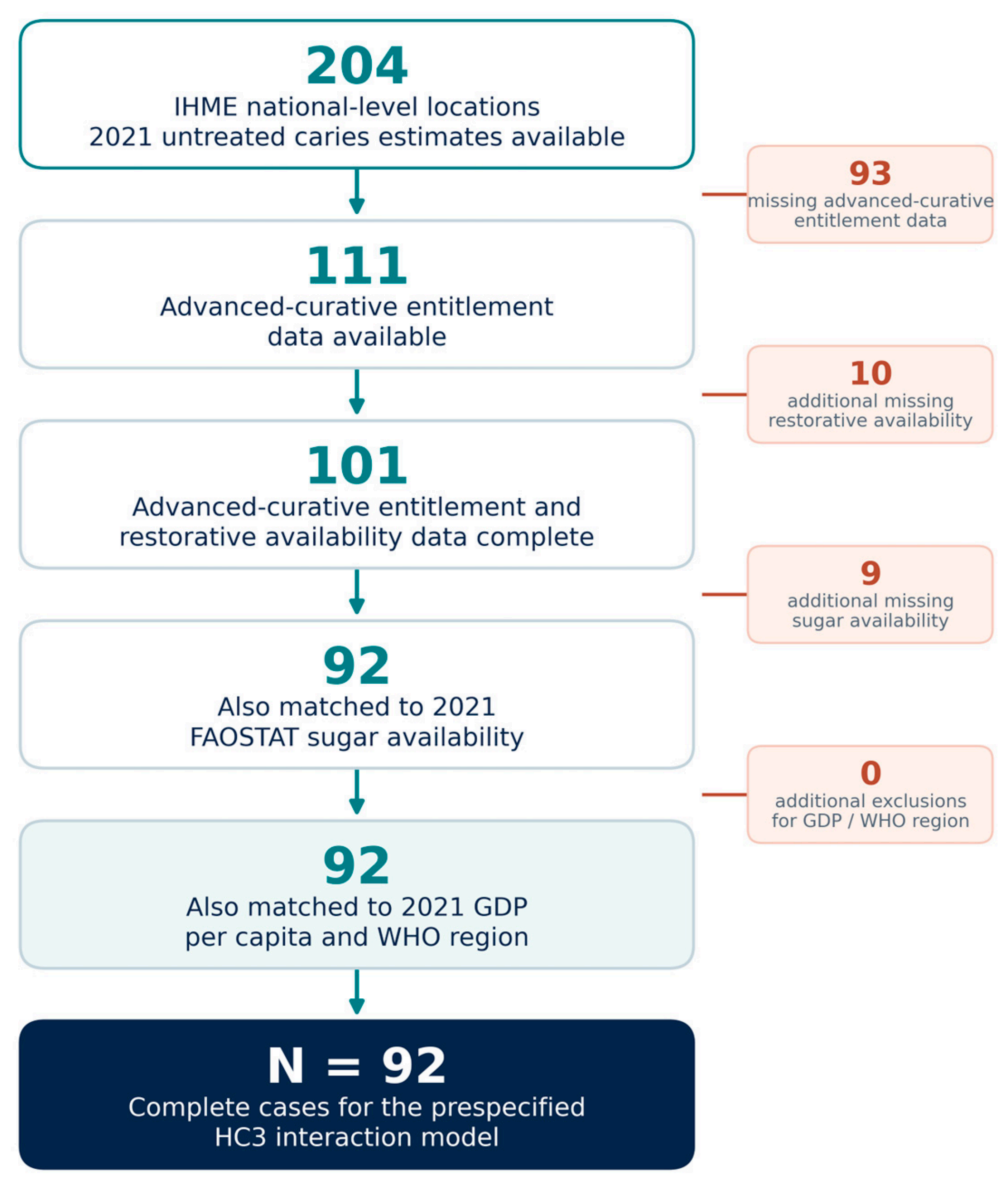
Flow of countries through data harmonization and primary analytical sample selection.

Of 204 initial national-level IHME locations, 93 lacked advanced-curative entitlement data, leaving 111; another 10 lacked restorative-availability data, leaving 101; and nine lacked 2021 sugar data. GDP and WHO-region information caused no further exclusions, yielding 92 countries (Figure 1; Supplementary Table S4). The joint categories comprised 49 Yes/Available, 14 Yes/Unavailable, 16 No/Available, and 13 No/Unavailable countries (Table 1).

**Figure 2:**
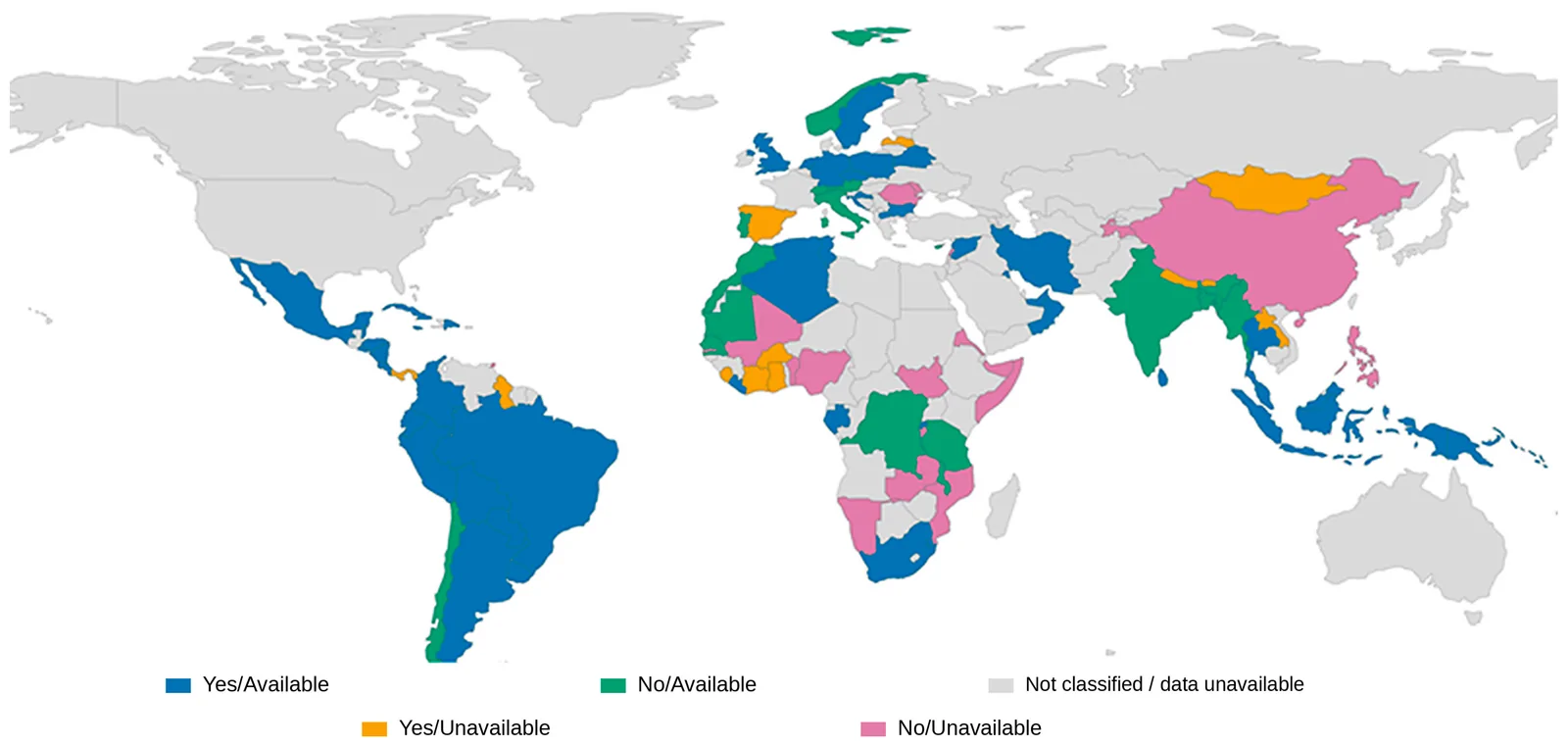
Geographic distribution of the four joint categories of advanced-curative entitlement and basic restorative service availability among 101 countries with observed exposure data.

Figure 2 shows the four joint categories among 101 countries with both exposure indicators; Supplementary Figure S1 summarizes WHO-region distributions. In the 92-country primary sample, crude caries prevalence varied across categories (Welch *F*[3, 28.80] = 4.92; *p* = 0.007), ranging from 39.72% (No/Available) to 47.09% (Yes/Available) (Table 1).

The crude Yes/Available versus No/Available difference was 7.36 pp (95% simultaneous CI, 2.05–12.67; Games–Howell *p* = 0.004); other pairwise comparisons were not statistically significant (Supplementary Table S5).

Descriptive comparisons between included and excluded locations showed no statistically significant differences in untreated caries prevalence (*p* = 0.590), GDP per capita (*p* = 0.149), or sugar availability (*p* = 0.894). However, inclusion differed by WHO region (permutation χ^2^ = 11.66; Cramér’s V = 0.245; *p* = 0.038) and by observed GDP-per-capita quartile (Pearson χ^2^ = 9.27; *p* = 0.026). Only 15 of 49 locations (30.6%) in the highest GDP quartile were included in the primary analysis. Detailed geographical and economic distributions by exclusion stage are provided in Supplementary Tables S3 and S6. These exploratory findings suggest nonuniform sample inclusion but do not establish the mechanism or direction of potential selection bias.

### 3.2. Country-Level Associations of Entitlement and Restorative Service Availability with Untreated Caries

The adjusted primary model showed an entitlement-by-availability interaction (β = 9.00 pp; 95% CI, 1.71–16.29; *p* = 0.016). When advanced-curative entitlement was absent, restorative availability was associated with lower caries prevalence (β = −6.62 pp; 95% CI, −12.23 to −1.02; *p* = 0.021); when entitlement was present, the corresponding conditional association was not statistically significant (β = 2.37 pp; 95% CI, −1.73 to 6.48; *p* = 0.253). Entitlement was not significantly associated with prevalence when restorative services were unavailable (β = −2.71 pp; 95% CI, −7.88 to 2.46; *p* = 0.301) (Table 2).

**Figure 3:**
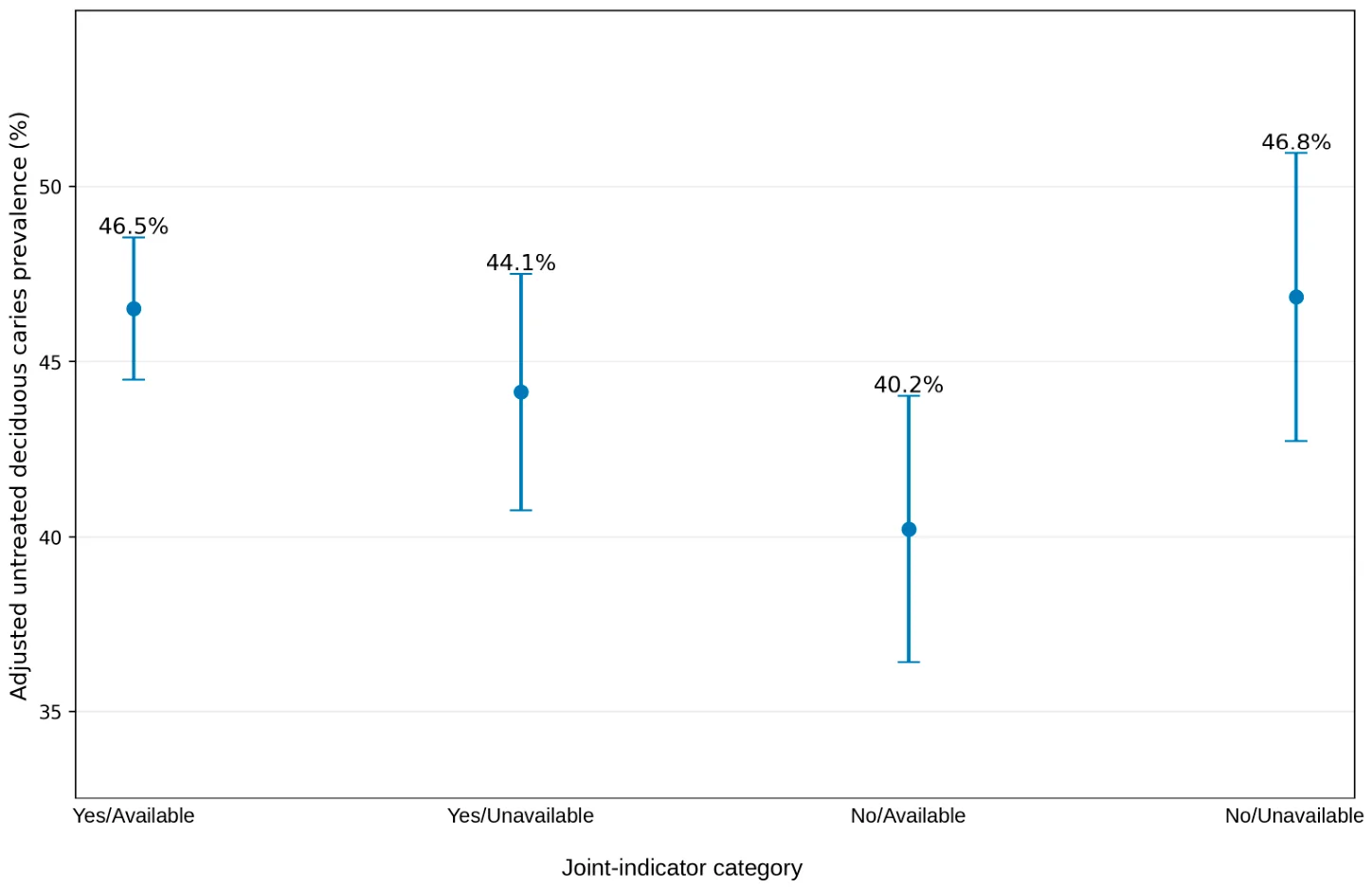
Adjusted predicted prevalence of untreated caries in the primary dentition across the four joint-indicator categories. Points represent marginally standardized predicted prevalence estimates from the prespecified HC3 interaction model; error bars represent 95% confidence intervals.

Marginally standardized estimates showed adjusted untreated caries prevalences of 46.51% (95% CI, 44.48–48.53) for the Yes/Available category, 44.13% (95% CI, 40.76–47.50) for Yes/Unavailable, 40.22% (95% CI, 36.41–44.02) for No/Available, and 46.84% (95% CI, 42.73–50.95) for No/Unavailable. Compared with the Yes/Available reference category, the No/Available category had a 6.29 pp lower adjusted predicted prevalence (95% CI, −11.06 to −1.52; Holm-adjusted *p* = 0.031). Neither the Yes/Unavailable nor the No/Unavailable category differed significantly from the reference category after multiplicity correction (Table 3 and Figure 3).

The model explained 43.7% of outcome variation (adjusted R^2^ = 0.368) and was statistically significant based on HC3-robust inference (*F*[10, 81] = 10.90; *p* < 0.001). WHO region was significantly associated with prevalence (*F*[5, 81] = 14.55; *p* < 0.001), whereas sugar availability was not (*p* = 0.300), and GDP showed a borderline inverse association (*p* = 0.056). Diagnostic tests indicated heteroskedasticity (*p* = 0.036) but no evidence of model misspecification according to the RESET test (*p* = 0.120); the maximum non-intercept VIF was 6.16 for the interaction term.

### 3.3. Sensitivity Analyses

The primary entitlement-by-availability interaction was positive in the HC3 OLS model (N = 92; β = 9.00 pp; 95% CI, 1.71–16.29; *p* = 0.016). The interaction remained positive after additional adjustment for dentist density using the nearest available observation from 2019 to 2023 (N = 88; β = 8.60 pp; 95% CI, 1.15–16.04; *p* = 0.024), restriction to countries with exact 2021 dentist-density observations (N = 65; β = 9.17 pp; 95% CI, 0.16–18.18; *p* = 0.046), and exclusion of seven countries exceeding the Cook’s distance threshold (N = 85; β = 7.26 pp; 95% CI, 1.46–13.06; *p* = 0.015; Supplementary Tables S7 and S8). The corresponding influence diagnostics, including Cook’s distance, leverage, and externally studentized residuals, are presented in Supplementary Figure S2A,B.

In the alternative specification using essential-curative entitlement instead of advanced-curative entitlement, the interaction remained positive (N = 92; β = 8.30 pp; 95% CI, 0.24–16.36; *p* = 0.044). Although this finding was consistent with the primary analysis in direction, the alternative entitlement indicator was not an exact clinical equivalent of the basic restorative service availability measure. Beta regression also yielded a positive interaction (N = 92; β = 0.372; 95% CI, 0.109–0.634; *p* = 0.005), although this coefficient was estimated on the logit-mean scale rather than the percentage-point scale. The child-population-weighted least-squares analysis produced a smaller interaction estimate (N = 92; β = 6.11 pp; 95% CI, −1.01 to 13.24; *p* = 0.092) than the primary unweighted model (N = 92; β = 9.00 pp; 95% CI, 1.71–16.29; *p* = 0.016). The weighted confidence interval included zero, indicating that the interaction was not statistically significant under child-population weighting. Thus, despite consistently positive interaction estimates, the strength of statistical evidence was sensitive to the analytical weighting scheme (Supplementary Table S7).

#### Exploratory Age-Specific and Age-Composition Analyses

Exploratory age-specific analyses were conducted using the same 92-country analytical sample and adjustment variables as the primary model. The interaction between advanced-curative entitlement and basic restorative service availability was positive for children aged 2–4 years (β = 14.18 pp; 95% CI, 2.76–25.60; *p* = 0.016) and for children aged 5–9 years (β = 5.91 pp; 95% CI, 0.14–11.68; *p* = 0.045). The interaction estimate was numerically larger in the younger age group, although a formal statistical comparison between the age-specific estimates was not performed. After additional adjustment for the proportion of children aged 5–9 years within the national population aged 1–9 years, the interaction estimate for the aggregated outcome remained similar to that of the primary model (β = 8.75 pp; 95% CI, 1.74–15.76; *p* = 0.015). These exploratory analyses were not prespecified and should not be interpreted as confirmatory evidence of age-specific differences (Table 4).

### 3.4. Secondary Entitlement Models

Neither routine/preventive entitlement (β = 2.33 pp; 95% CI, −1.34 to 5.99; *p* = 0.211) nor essential-curative entitlement (β = 3.15 pp; 95% CI, −0.44 to 6.73; *p* = 0.085) was significantly associated with prevalence in separate adjusted models (Supplementary Table S9).

## 4. Discussion

In this 92-country ecological study, the association of reported basic restorative availability with untreated primary-tooth caries differed by advanced-curative entitlement. Among countries without advanced-curative entitlement, service availability was associated with lower prevalence; the conditional association was not statistically significant among countries with entitlement. No/Available also had a lower adjusted predicted prevalence than Yes/Available. These findings describe associations between clinically non-equivalent indicators, not the effectiveness of any policy configuration.

These findings highlight the need to distinguish formal entitlement, reported service availability, actual service delivery, and effective access to care when monitoring progress toward universal oral-health coverage. The first two indicators do not directly establish whether children receive treatment. The WHO Universal Health Coverage framework similarly distinguishes who is covered, which services are included, and the extent to which costs are covered [21]. Comparative oral-health research has also emphasized that legal or nominal eligibility does not necessarily translate into effective access. McAuliffe et al., for example, showed that broad formal coverage for children may coexist with barriers to publicly funded care and substantial private-sector involvement [12]. Henschke et al. likewise reported substantial variation in the organization and financing of dental coverage across countries and cautioned that oral-health outcomes cannot be directly inferred from the breadth of public coverage alone [15]. The present ecological findings add to this body of literature by indicating that the country-level association between advanced-curative entitlement and untreated caries prevalence differed according to the reported national availability of basic restorative services.

The unexpectedly lower prevalence in No/Available countries is not evidence that absent advanced-curative entitlement is beneficial. A negative response for advanced-care coverage may coexist with basic restorative coverage or alternative public/private financing. Differences in past disease burden, preventive programs, fluoride exposure, socioeconomic circumstances, and treatment access—and policies introduced in response to need—could also explain the association [13,22]. Cross-sectional data cannot distinguish these explanations or demonstrate policy–service alignment.

Secondary entitlement models showed no independent associations for routine/preventive or essential-curative entitlement. Binary national benefit indicators may poorly capture intervention intensity, coverage, quality, or actual use [16]. By contrast, Foote et al. reported an association between preventive policy and dental outcomes in older children across 19 OECD countries, but studied different ages, outcomes, and policy measures [14]. The findings therefore are not evidence against preventive or essential care.

WHO region was associated with untreated caries, while sugar availability was not and GDP had a borderline inverse association. This ecological sugar measure describes food supply, not children’s free-sugar consumption patterns; its null result does not challenge evidence linking sugar intake with caries [6,23]. Residual regional differences may reflect preventive programs, fluoride exposure, inequality, and health-system organization.

Interaction estimates remained positive across sensitivity analyses, but the interaction was no longer statistically significant after child-population weighting. This finding indicates that the statistical evidence was sensitive to the weighting scheme. Dentist-density sensitivity models also involved smaller, compositionally different samples, limiting direct comparability with the primary model.

The exploratory age-specific analyses provided additional information regarding the consistency of the observed country-level associations across developmental stages. Positive interaction estimates were observed in both the 2–4-year and 5–9-year age groups, although the estimated interaction was numerically larger among younger children. This difference should be interpreted cautiously because the age-specific interaction estimates were not formally compared and the analyses were exploratory. Furthermore, the aggregated 1–9-year prevalence reflected the age composition of each country’s child population rather than a standardized age distribution. Differences in population age structure may therefore contribute to cross-country variation in untreated caries prevalence. Adjustment for the proportion of children aged 5–9 years resulted in only a small change in the interaction estimate (from 9.00 to 8.75 pp), suggesting that this measured aspect of age composition did not substantially alter the primary association. Nevertheless, this adjustment does not account for all potential age-related differences between countries, and the findings should not be interpreted as excluding residual age-composition effects.

Several limitations should be considered. The ecological and cross-sectional design precludes individual-level or causal inference. Although the principal indicators refer to 2021, untreated caries may reflect earlier healthcare conditions, and historical policy implementation dates were unavailable. Therefore, temporal precedence could not be established, reverse causality could not be ruled out, and lagged analyses were not feasible (Supplementary Table S10). Furthermore, advanced-curative entitlement and basic restorative service availability represent clinically non-equivalent levels of care [19,20]. Their combinations cannot establish policy–service concordance, implementation gaps, effective access, utilization, or care quality, and essential-curative entitlement was not a procedure-matched alternative.

Residual country-level confounding may persist despite adjustment for GDP, sugar availability, WHO region, and dentist density in sensitivity analyses. Potential unmeasured factors include fluoride exposure, preventive programs, oral-health expenditure, education, socioeconomic inequality, and geographic access. Moreover, national GDP obscures within-country disparities, while food availability does not measure individual dietary exposure. Only 92 of 204 locations (45.1%) had complete primary data, mainly because of missing entitlement information. Inclusion varied by WHO region and GDP quartile, limiting generalizability, while unequal joint-category sizes (49, 14, 16, and 13 countries) may reduce precision. Excluding influential observations does not resolve these concerns.

Finally, the aggregated 1–9-year outcome was derived from modeled GBD age-specific estimates. Because joint posterior draws and correlations between age groups were unavailable, uncertainty in the underlying estimates could not be propagated into the regression confidence intervals. No independent age-standardized estimate for children aged 1–9 years was available for external validation. Differences in national age composition may therefore still influence country-level comparisons despite exploratory age-specific analyses and age-composition adjustment.

## 5. Conclusions

Advanced-curative entitlement modified the country-level association of reported basic restorative availability with untreated caries prevalence in primary teeth among children aged 1–9 years. The lower adjusted prevalence in No/Available countries does not imply a benefit from absent entitlement or demonstrate policy–service alignment. The interaction was not statistically significant after child-population weighting. Longitudinal analyses using clinically comparable indicators and measures of accessibility and actual treatment are needed before drawing policy-effect conclusions.

## Figures and Tables

**Table 1:** Characteristics of the primary analytical sample overall and by joint categories of advanced-curative entitlement and basic restorative service availability.

Characteristic	Overall	Yes/Available	Yes/Unavailable	No/Available	No/Unavailable	Test Statistic	Effect Size	*p* Value
Countries, *n*	92	49	14	16	13			
Untreated caries prevalence (%), mean ± SD	45.10 ± 7.60	47.09 ± 6.71	43.46 ± 6.97	39.72 ± 6.71	45.98 ± 9.49	*F*(3, 28.80) = 4.92 ^a^	-	**0.007**
GDP per capita (US$), median [IQR]	5800.29 [2515.25–15,058.48]	6828.76 [4010.76–12,962.27]	3048.33 [1802.09–14,097.57]	3134.39 [1509.26–33,916.51]	3971.44 [1127.16–5274.61]	H(3) = 5.56 ^b^	0.029 ^c^	0.135
Sugar availability (g/person/day), mean ± SD	67.35 ± 32.96	77.65 ± 27.88	44.72 ± 29.39	59.14 ± 36.70	62.98 ± 37.01	F(3, 28.21) = 5.01 ^a^	-	**0.007**
Dentists per 10,000, median [IQR]	88; 2.31 [0.69–7.25]	47; 3.58 [1.10–7.69]	13; 0.97 [0.40–4.86]	16; 1.71 [0.23–7.62]	12; 1.99 [0.20–3.52]	H(3) = 4.13 ^b^	0.014 ^c^	0.247
WHO region, *n* (%)	N = 92					χ^2^(15) = 20.26 ^d^	0.271 ^e^	0.161
Africa	23 (25.0%)	7 (14.3%)	5 (35.7%)	5 (31.2%)	6 (46.2%)			
Americas	20 (21.7%)	16 (32.7%)	2 (14.3%)	1 (6.2%)	1 (7.7%)			
Eastern Mediterranean	8 (8.7%)	6 (12.2%)	0 (0.0%)	1 (6.2%)	1 (7.7%)			
Europe	22 (23.9%)	10 (20.4%)	3 (21.4%)	6 (37.5%)	3 (23.1%)			
South-East Asia	9 (9.8%)	4 (8.2%)	2 (14.3%)	3 (18.8%)	0 (0.0%)			
Western Pacific	10 (10.9%)	6 (12.2%)	2 (14.3%)	0 (0.0%)	2 (15.4%)			

GDP = gross domestic product; IQR = interquartile range; SD = standard deviation. ^a^ Welch analysis of variance; ^b^ Kruskal–Wallis test; ^c^ ε^2^ effect size; ^d^ Pearson chi square test; ^e^ Cramér’s V. Bold *p* values indicate statistical significance (*p* < 0.05).

**Table 2:** Primary multivariable model of advanced-curative entitlement, basic restorative service availability, their interaction, and untreated caries prevalence.

Term	β	95% CI	Test Statistic	*p* Value
Intercept	53.037	39.248 to 66.826	7.65	**<0.001**
WHO region: Americas vs. Africa	7.365	3.326 to 11.403	3.63	**<0.001**
WHO region: Eastern Mediterranean vs. Africa	8.784	3.171 to 14.397	3.11	**0.003**
WHO region: Europe vs. Africa	11.020	5.282 to 16.758	3.82	**<0.001**
WHO region: South-East Asia vs. Africa	12.080	7.790 to 16.370	5.60	**<0.001**
WHO region: Western Pacific vs. Africa	12.721	9.324 to 16.117	7.45	**<0.001**
Advanced-curative entitlement: Yes vs. No	−2.707	−7.877 to 2.462	−1.04	0.301
Restorative service availability: Available vs. Unavailable	−6.623	−12.226 to −1.020	−2.35	**0.021**
Advanced-curative entitlement × restorative service availability	8.997	1.706 to 16.289	2.46	**0.016**
ln(GDP per capita)	−1.776	−3.597 to 0.045	−1.94	0.056
Sugar availability per 10 g/day	0.250	−0.226 to 0.726	1.04	0.300

N = 92; HC3-robust standard errors. β coefficients indicate percentage-point (pp) differences in untreated caries prevalence; Africa is the reference WHO region. Because the model includes an interaction, the restorative availability coefficient applies when entitlement = No, and the entitlement coefficient when services = Unavailable. CI = confidence interval; GDP = gross domestic product; ln = natural logarithm. Bold *p* values indicate *p* < 0.05.

**Table 3:** Adjusted predicted prevalence of untreated caries and prespecified contrasts across the four joint-indicator categories.

Joint-Indicator Category	Adjusted Predicted Prevalence, % (95% CI)	Difference vs. Yes/Available, pp (95% CI)	Holm-Adjusted *p*
Yes/Available	46.51 (44.48 to 48.53)	Reference	—
Yes/Unavailable	44.13 (40.76 to 47.50)	−2.37 (−6.48 to 1.73)	0.507
No/Available	40.22 (36.41 to 44.02)	−6.29 (−11.06 to −1.52)	**0.031**
No/Unavailable	46.84 (42.73 to 50.95)	0.33 (−4.31 to 4.98)	0.887

Estimates were obtained by marginal standardization from the prespecified HC3 interaction model. Yes/Available was the reference category; Holm correction was applied to the three contrasts. CI = confidence interval; pp = percentage points. Bold *p* values indicate *p* < 0.05.

**Table 4:** Exploratory age-specific and age-composition analyses of the entitlement-by-availability interaction.

Outcome/Model	Interaction β (95% CI), pp	*p* Value
Ages 1–9 years (primary model)	9.00 (1.71–16.29)	**0.016**
Ages 2–4 years (exploratory)	14.18 (2.76–25.60)	**0.016**
Ages 5–9 years (exploratory)	5.91 (0.14–11.68)	**0.045**
Ages 1–9 years, age-composition-adjusted (exploratory)	8.75 (1.74–15.76)	**0.015**

All models used HC3-robust OLS adjusted for ln(GDP per capita), sugar availability, and WHO region (N = 92). The age-composition model additionally adjusted for the proportion aged 5–9 years. Only the primary model was prespecified; exploratory *p* values were not adjusted for multiple testing. CI = confidence interval; pp = percentage points. Bold *p* values indicate *p* < 0.05.

## Data Availability

The harmonized country-level analytical dataset is provided in Supplementary Table S2. Source data are publicly available from the Institute for Health Metrics and Evaluation, the World Health Organization Global Health Observatory, FAOSTAT, and the World Bank World Development Indicators.

## Supplementary Materials

The following supporting information can be downloaded at: https://www.mdpi.com/article/10.3390/healthcare14213609/s1, Supplementary Table S1: Data sources, availability, and analytical role; Supplementary Table S2: Harmonized country-level analytical dataset; Supplementary Table S3: Assessment of missingness and analytical-sample selection; Supplementary Table S4: Sequential country selection and variable-level missingness; Supplementary Table S5: Games–Howell post hoc comparisons of crude untreated caries prevalence across joint-indicator categories; Supplementary Table S6: Geographical and economic distribution of included and excluded locations; Supplementary Table S7: Prespecified sensitivity and robustness analyses for the primary entitlement-by-availability interaction; Supplementary Table S8: Country-level influence diagnostics for observations exceeding prespecified screening thresholds in the primary regression model; Supplementary Table S9: Secondary entitlement models for untreated deciduous caries prevalence; Supplementary Table S10: Temporal interpretation of study indicators; Supplementary Figure S1: Distribution of joint-indicator categories across WHO regions among 101 countries with observed entitlement and restorative-service data; Supplementary Figure S2A: Cook’s distance for the primary regression model; Supplementary Figure S2B: Leverage and externally studentized residual diagnostics.

## Abbreviations

The following abbreviations are used:

CI	Confidence interval
ECC	Early childhood caries
GBD	Global Burden of Disease
HC3	HC3 heteroskedasticity-robust covariance estimator
IHME	Institute for Health Metrics and Evaluation
ISO3	ISO alpha-3 country code
pp	Percentage points
SD	Standard deviation
STROBE	Strengthening the Reporting of Observational Studies in Epidemiology
UHC	Universal health coverage
WHO	World Health Organization
