# Supplementary material for: Advanced-Curative Oral-Health Entitlement, Basic Restorative Service Availability, and Untreated Dental Caries in Primary Dentition: A Cross-National Ecological Study
Source: Healthcare (Basel). 2026 Oct 25;14(21):3609. doi: 10.3390/healthcare14213609 (PMC13762583; doi:10.3390/healthcare14213609)
Supplement: Supplementary file 1 [file healthcare-14-03609-s001.zip › healthcare-4610437-supplementary.pdf]

Supplementary Materials

Supplementary Tables

Supplementary Table S1. Data sources, availability, and analytical role

Indicator	Reference year	Source	Available national/location records	Role in analysis
Untreated caries of deciduous teeth, ages 1–9 years	2021	IHME GBD 2023 release	204 national-level locations	Primary outcome
Advanced curative oral/dental care in health-benefit package	2021	WHO GHO	111 countries	Primary policy exposure
Essential curative oral health care in health-benefit package	2021	WHO GHO	111 countries	Secondary / alternative construct
Routine and preventive oral health care in health-benefit package	2021	WHO GHO	111 countries	Secondary exposure
Basic restorative dental procedures availability	2021	WHO GHO	176 countries	Primary service exposure
Sugar (Raw Equivalent), food supply quantity	2021	FAOSTAT Food Balances	176 ISO3-mappable national entries	Core covariate
GDP per capita (current US$), NY.GDP.PCAP.CD	2021	World Bank WDI	209 non-missing ISO3 country/territory records	Core covariate; ln-transformed
Dentists per 10,000 population	2021 / nearest 2019–2023	WHO GHO	193 overall; 172 in 2019–2023; 116 exact 2021	Sensitivity analysis

Source-specific availability counts are reported before complete-case exclusions and do not necessarily refer to the same set of 204 IHME outcome locations. The non-country FAOSTAT China aggregate was excluded; mainland China was retained. World Bank aggregates and footer rows were excluded before ISO3 matching.

Supplementary Table S2. Harmonized country-level analytical dataset

Country	ISO3	WHO region	Caries %	Advanced	Essential	Preventive	Restorative	Profile	GDP 2021	Sugar g/day	Dentists/10k (year)	Primary
Afghanistan	AFG	Eastern Mediterranean	41.94	—	—	—	Available	—	356	35.04	0.06 (2020)	Excluded
Albania	ALB	Europe	54.33	—	—	—	—	—	7,242	54.05	—	Excluded
Algeria	DZA	Africa	44.99	Yes	Yes	Yes	Available	Yes/Available	4,161	78.71	3.58 (2020)	Included
American Samoa	ASM	—	48.88	—	—	—	—	—	15,236	—	—	Excluded
Andorra	AND	Europe	41.59	—	—	—	Available	—	42,426	—	11.61 (2021)	Excluded
Angola	AGO	Africa	32.93	—	—	—	—	—	2,267	28.08	0.07 (2022)	Excluded
Antigua and Barbuda	ATG	Americas	48.02	—	—	—	Available	—	17,349	52.30	—	Excluded
Argentina	ARG	Americas	47.36	Yes	Yes	Yes	Available	Yes/Available	10,738	96.79	11.85 (2021)	Included
Armenia	ARM	Europe	55.03	—	—	—	Available	—	4,685	70.11	2.93 (2021)	Excluded
Australia	AUS	Western Pacific	56.35	—	—	—	Available	—	60,759	90.44	6.20 (2021)	Excluded
Austria	AUT	Europe	39.82	No	Yes	Yes	Available	No/Available	53,649	87.37	6.16 (2021)	Included
Azerbaijan	AZE	Europe	54.25	—	—	—	Available	—	5,408	64.55	3.22 (2021)	Excluded
Bahamas	BHS	Americas	46.71	—	—	—	Unavailable	—	30,368	72.90	—	Excluded
Bahrain	BHR	Eastern Mediterranean	44.06	—	—	—	Available	—	27,148	117.12	0.80 (2020)	Excluded
Bangladesh	BGD	South-East Asia	48.74	No	No	No	Available	No/Available	2,483	17.86	0.69 (2021)	Included
Barbados	BRB	Americas	48.44	—	—	—	Unavailable	—	21,084	131.81	2.45 (2021)	Excluded
Belarus	BLR	Europe	59.30	Yes	Yes	Yes	Available	Yes/Available	7,490	87.59	5.79 (2021)	Included
Belgium	BEL	Europe	37.74	Yes	Yes	Yes	—	—	51,658	125.40	7.71 (2021)	Excluded
Belize	BLZ	Americas	47.50	Yes	Yes	Yes	Available	Yes/Available	6,143	93.45	1.72 (2021)	Included
Benin	BEN	Africa	38.66	No	No	Yes	Unavailable	No/Unavailable	1,319	—	0.04 (2021)	Excluded
Bermuda	BMU	—	49.05	—	—	—	—	—	112,765	—	—	Excluded
Bhutan	BTN	South-East Asia	39.48	Yes	Yes	Yes	Unavailable	Yes/Unavailable	3,571	38.96	0.97 (2021)	Included
Bolivia (Plurinational State of)	BOL	Americas	45.80	Yes	Yes	Yes	Available	Yes/Available	4,011	51.40	2.29 (2021)	Included
Bosnia and Herzegovina	BIH	Europe	58.87	—	—	—	Available	—	7,295	63.53	2.58 (2019)	Excluded
Botswana	BWA	Africa	38.15	—	—	—	Unavailable	—	7,808	65.23	0.37 (2021)	Excluded
Brazil	BRA	Americas	44.56	Yes	Yes	Yes	Available	Yes/Available	7,973	94.14	6.83 (2021)	Included
Brunei Darussalam	BRN	Western Pacific	50.95	—	—	—	Available	—	31,007	—	2.61 (2021)	Excluded
Bulgaria	BGR	Europe	54.62	Yes	Yes	Yes	Available	Yes/Available	12,967	63.04	10.99 (2021)	Included
Burkina Faso	BFA	Africa	32.69	Yes	Yes	Yes	Unavailable	Yes/Unavailable	896	12.55	0.05 (2021)	Included
Burundi	BDI	Africa	35.07	No	No	No	Unavailable	No/Unavailable	264	—	0.01 (2021)	Excluded

Country	ISO3	WHO region	Caries %	Advanced	Essential	Preventive	Restorative	Profile	GDP 2021	Sugar g/day	Dentists/10k (year)	Primary
Cabo Verde	CPV	Africa	42.20	No	Yes	Yes	Unavailable	No/Unavailable	3,971	40.49	2.33 (2019)	Included
Cambodia	KHM	Western Pacific	50.76	—	—	—	Available	—	2,167	17.45	—	Excluded
Cameroon	CMR	Africa	38.82	—	—	—	Available	—	1,672	23.32	0.05 (2021)	Excluded
Canada	CAN	Americas	37.95	—	—	—	Available	—	52,887	97.64	6.53 (2020)	Excluded
Central African Republic	CAF	Africa	32.39	—	—	—	Available	—	492	—	0.00 (2021)	Excluded
Chad	TCD	Africa	38.53	Yes	Yes	Yes	—	—	946	—	0.01 (2021)	Excluded
Chile	CHL	Americas	50.83	No	Yes	No	Available	No/Available	16,216	115.40	14.83 (2021)	Included
China	CHN	Western Pacific	52.34	No	No	Yes	Unavailable	No/Unavailable	12,887	18.05	—	Included
Colombia	COL	Americas	40.94	Yes	Yes	Yes	Available	Yes/Available	6,223	87.70	8.40 (2021)	Included
Comoros	COM	Africa	43.59	Yes	Yes	Yes	Unavailable	Yes/Unavailable	1,588	30.16	0.40 (2020)	Included
Congo	COG	Africa	40.96	—	—	—	Available	—	2,516	27.21	0.02 (2022)	Excluded
Cook Islands	COK	Western Pacific	52.39	—	—	—	Available	—	—	—	3.73 (2019)	Excluded
Costa Rica	CRI	Americas	44.80	Yes	Yes	Yes	Available	Yes/Available	12,962	114.16	10.73 (2021)	Included
Croatia	HRV	Europe	56.41	Yes	Yes	Yes	Available	Yes/Available	17,789	81.40	9.25 (2021)	Included
Cuba	CUB	Americas	46.90	Yes	Yes	Yes	Available	Yes/Available	—	—	18.79 (2021)	Excluded
Cyprus	CYP	Europe	38.66	No	No	No	Available	No/Available	32,938	45.56	7.58 (2021)	Included
Czechia	CZE	Europe	56.77	Yes	Yes	Yes	Available	Yes/Available	27,696	85.81	7.56 (2021)	Included
Côte d'Ivoire	CIV	Africa	38.51	Yes	Yes	Yes	Unavailable	Yes/Unavailable	2,456	27.32	0.15 (2021)	Included
Democratic People's Republic of Korea	PRK	South-East Asia	46.70	—	—	—	Available	—	—	—	—	Excluded
Democratic Republic of the Congo	COD	Africa	32.92	No	No	Yes	Available	No/Available	596	7.64	0.03 (2021)	Included
Denmark	DNK	Europe	25.27	—	—	—	Available	—	69,341	106.05	7.18 (2021)	Excluded
Djibouti	DJI	Eastern Mediterranean	34.98	—	—	—	Available	—	3,026	94.74	—	Excluded
Dominica	DMA	Americas	48.33	—	—	—	Unavailable	—	8,376	—	—	Excluded
Dominican Republic	DOM	Americas	48.03	Yes	Yes	Yes	Available	Yes/Available	8,527	107.15	2.21 (2021)	Included
Ecuador	ECU	Americas	47.33	Yes	Yes	Yes	Available	Yes/Available	6,061	58.66	2.97 (2020)	Included
Egypt	EGY	Eastern Mediterranean	44.70	—	—	—	—	—	3,827	65.29	3.09 (2021)	Excluded
El Salvador	SLV	Americas	44.61	Yes	Yes	Yes	Available	Yes/Available	4,643	83.42	1.45 (2021)	Included
Equatorial Guinea	GNQ	Africa	32.98	—	—	—	Available	—	6,946	—	—	Excluded
Eritrea	ERI	Africa	35.32	No	Yes	Yes	Unavailable	No/Unavailable	—	—	0.18 (2021)	Excluded
Estonia	EST	Europe	59.74	—	—	—	Available	—	27,951	52.27	10.11 (2021)	Excluded

Country	ISO3	WHO region	Caries %	Advanced	Essential	Preventive	Restorative	Profile	GDP 2021	Sugar g/day	Dentists/10k (year)	Primary
Eswatini	SWZ	Africa	43.93	Yes	No	No	Available	Yes/Available	3,984	57.23	0.18 (2020)	Included
Ethiopia	ETH	Africa	45.27	No	No	No	—	—	893	22.11	0.16 (2020)	Excluded
Fiji	FJI	Western Pacific	50.00	—	—	—	Available	—	4,537	81.73	1.17 (2019)	Excluded
Finland	FIN	Europe	41.19	—	—	—	Available	—	53,099	80.90	7.19 (2021)	Excluded
France	FRA	Europe	36.11	—	—	—	Available	—	43,725	96.03	6.79 (2021)	Excluded
Gabon	GAB	Africa	31.88	Yes	Yes	Yes	Available	Yes/Available	8,181	53.34	0.15 (2020)	Included
Gambia	GMB	Africa	40.22	No	No	No	Unavailable	No/Unavailable	782	128.60	0.04 (2021)	Included
Georgia	GEO	Europe	61.30	—	—	—	Available	—	5,084	97.95	6.50 (2021)	Excluded
Germany	DEU	Europe	29.04	Yes	Yes	Yes	Available	Yes/Available	52,349	102.66	8.52 (2021)	Included
Ghana	GHA	Africa	39.43	Yes	Yes	Yes	Unavailable	Yes/Unavailable	2,445	32.77	—	Included
Greece	GRC	Europe	24.77	—	—	—	Available	—	20,653	85.40	13.01 (2021)	Excluded
Greenland	GRL	—	44.61	—	—	—	—	—	58,136	—	—	Excluded
Grenada	GRD	Americas	48.14	—	—	—	Available	—	9,617	83.95	—	Excluded
Guam	GUM	—	52.09	—	—	—	—	—	38,075	—	—	Excluded
Guatemala	GTM	Americas	43.47	—	—	—	Unavailable	—	4,913	144.19	2.59 (2020)	Excluded
Guinea	GIN	Africa	38.72	—	—	—	Unavailable	—	1,245	50.68	0.03 (2022)	Excluded
Guinea-Bissau	GNB	Africa	38.90	—	—	—	—	—	927	6.27	0.01 (2021)	Excluded
Guyana	GUY	Americas	47.39	Yes	Yes	Yes	Unavailable	Yes/Unavailable	9,861	73.78	0.68 (2020)	Included
Haiti	HTI	Americas	44.41	—	—	—	Unavailable	—	1,825	62.33	—	Excluded
Honduras	HND	Americas	42.58	Yes	Yes	Yes	Available	Yes/Available	2,735	90.66	7.10 (2023)	Included
Hungary	HUN	Europe	56.95	—	—	—	Available	—	19,031	74.22	7.10 (2021)	Excluded
Iceland	ISL	Europe	40.15	Yes	Yes	Yes	—	—	70,425	70.05	7.89 (2021)	Excluded
India	IND	South-East Asia	43.87	No	Yes	Yes	Available	No/Available	2,240	47.42	2.06 (2021)	Included
Indonesia	IDN	Western Pacific	53.36	Yes	Yes	Yes	Available	Yes/Available	4,287	70.11	1.23 (2021)	Included
Iran (Islamic Republic of)	IRN	Eastern Mediterranean	51.93	Yes	Yes	Yes	Available	Yes/Available	4,605	72.38	5.26 (2023)	Included
Iraq	IRQ	Eastern Mediterranean	44.66	—	—	—	Available	—	4,868	57.92	4.36 (2021)	Excluded
Ireland	IRL	Europe	43.57	—	—	—	Available	—	103,783	86.60	4.63 (2021)	Excluded
Israel	ISR	Europe	53.02	—	—	—	Available	—	52,258	89.32	11.56 (2021)	Excluded
Italy	ITA	Europe	39.45	No	No	No	Available	No/Available	36,853	87.37	7.73 (2021)	Included
Jamaica	JAM	Americas	47.33	Yes	Yes	Yes	Available	Yes/Available	5,626	109.34	—	Included
Japan	JPN	Western Pacific	44.03	—	—	—	Available	—	41,581	—	8.25 (2020)	Excluded

Country	ISO3	WHO region	Caries %	Advanced	Essential	Preventive	Restorative	Profile	GDP 2021	Sugar g/day	Dentists/10k (year)	Primary
Jordan	JOR	Eastern Mediterranean	44.56	—	—	—	Available	—	4,582	88.82	8.04 (2021)	Excluded
Kazakhstan	KAZ	Europe	55.09	—	—	—	Available	—	9,984	75.51	2.30 (2021)	Excluded
Kenya	KEN	Africa	42.25	—	—	—	Unavailable	—	2,061	57.92	0.05 (2020)	Excluded
Kiribati	KIR	Western Pacific	49.29	—	—	—	Available	—	2,224	115.86	0.53 (2023)	Excluded
Kuwait	KWT	Eastern Mediterranean	46.06	Yes	Yes	Yes	Available	Yes/Available	34,019	74.19	9.18 (2021)	Included
Kyrgyzstan	KGZ	Europe	53.01	No	No	Yes	—	—	1,350	42.08	1.71 (2021)	Excluded
Lao People's Democratic Republic	LAO	Western Pacific	51.61	Yes	Yes	No	Unavailable	Yes/Unavailable	2,526	27.26	0.76 (2020)	Included
Latvia	LVA	Europe	54.92	Yes	Yes	Yes	Unavailable	Yes/Unavailable	20,262	79.48	7.15 (2021)	Included
Lebanon	LBN	Eastern Mediterranean	45.08	No	No	No	Unavailable	No/Unavailable	4,045	108.22	12.50 (2020)	Included
Lesotho	LSO	Africa	32.43	—	—	—	—	—	1,067	20.16	0.17 (2019)	Excluded
Liberia	LBR	Africa	38.72	Yes	Yes	Yes	Available	Yes/Available	668	19.01	0.01 (2021)	Included
Libya	LBY	Eastern Mediterranean	43.58	—	—	—	Available	—	4,936	70.05	5.80 (2020)	Excluded
Lithuania	LTU	Europe	62.40	—	—	—	Available	—	23,883	72.36	10.50 (2021)	Excluded
Luxembourg	LUX	Europe	40.34	—	—	—	Available	—	134,966	30.85	—	Excluded
Madagascar	MDG	Africa	37.00	—	—	—	Available	—	483	24.99	0.08 (2022)	Excluded
Malawi	MWI	Africa	43.69	No	Yes	Yes	Available	No/Available	617	27.21	0.08 (2020)	Included
Malaysia	MYS	Western Pacific	51.14	Yes	Yes	Yes	Available	Yes/Available	10,903	111.86	3.67 (2021)	Included
Maldives	MDV	South-East Asia	50.54	Yes	Yes	Yes	Available	Yes/Available	10,176	63.51	1.02 (2020)	Included
Mali	MLI	Africa	38.29	No	Yes	Yes	Unavailable	No/Unavailable	1,027	—	0.01 (2022)	Excluded
Malta	MLT	Europe	40.54	Yes	Yes	Yes	Unavailable	Yes/Unavailable	38,078	107.75	5.15 (2021)	Included
Marshall Islands	MHL	Western Pacific	50.64	—	—	—	Available	—	6,315	25.67	1.45 (2021)	Excluded
Mauritania	MRT	Africa	39.80	No	No	No	Available	No/Available	1,947	112.79	0.26 (2021)	Included
Mauritius	MUS	Africa	45.08	Yes	Yes	Yes	Available	Yes/Available	9,178	87.51	3.24 (2022)	Included
Mexico	MEX	Americas	47.79	Yes	Yes	Yes	Available	Yes/Available	10,314	94.00	1.18 (2021)	Included
Micronesia (Federated States of)	FSM	Western Pacific	50.62	Yes	Yes	Yes	Available	Yes/Available	3,508	57.01	—	Included
Monaco	MCO	Europe	39.52	—	—	—	—	—	223,823	—	9.19 (2020)	Excluded
Mongolia	MNG	Western Pacific	54.36	Yes	Yes	Yes	Unavailable	Yes/Unavailable	4,518	24.58	4.86 (2021)	Included
Montenegro	MNE	Europe	55.24	—	—	—	Available	—	9,316	57.04	0.60 (2021)	Excluded
Morocco	MAR	Eastern Mediterranean	44.63	No	No	Yes	Available	No/Available	3,786	85.59	1.36 (2021)	Included

Country	ISO3	WHO region	Caries %	Advanced	Essential	Preventive	Restorative	Profile	GDP 2021	Sugar g/day	Dentists/10k (year)	Primary
Mozambique	MOZ	Africa	34.44	No	Yes	Yes	Unavailable	No/Unavailable	510	24.58	0.11 (2021)	Included
Myanmar	MMR	South-East Asia	42.74	No	No	Yes	Available	No/Available	1,243	7.97	0.72 (2019)	Included
Namibia	NAM	Africa	33.43	No	Yes	No	Unavailable	No/Unavailable	4,413	66.27	0.57 (2021)	Included
Nauru	NRU	Western Pacific	50.73	—	—	—	Available	—	12,748	104.05	3.45 (2019)	Excluded
Nepal	NPL	South-East Asia	49.51	Yes	Yes	Yes	Unavailable	Yes/Unavailable	1,253	23.84	1.39 (2021)	Included
Netherlands	NLD	Europe	39.28	Yes	Yes	Yes	Available	Yes/Available	60,142	99.32	5.74 (2021)	Included
New Zealand	NZL	Western Pacific	24.56	—	—	—	Unavailable	—	49,902	120.14	5.07 (2021)	Excluded
Nicaragua	NIC	Americas	41.03	Yes	Yes	Yes	Available	Yes/Available	2,138	102.55	0.37 (2021)	Included
Niger	NER	Africa	41.52	—	—	—	Unavailable	—	610	14.96	0.01 (2022)	Excluded
Nigeria	NGA	Africa	37.65	No	No	Yes	Unavailable	No/Unavailable	2,787	24.36	0.22 (2021)	Included
Niue	NIU	Western Pacific	52.38	—	—	—	Available	—	—	—	—	Excluded
North Macedonia	MKD	Europe	54.75	Yes	Yes	Yes	Available	Yes/Available	7,621	110.88	8.77 (2020)	Included
Northern Mariana Islands	MNP	—	51.72	—	—	—	—	—	19,456	—	—	Excluded
Norway	NOR	Europe	37.53	No	No	No	Available	No/Available	96,443	78.27	8.63 (2021)	Included
Oman	OMN	Eastern Mediterranean	44.80	Yes	Yes	Yes	Available	Yes/Available	19,403	65.59	3.61 (2021)	Included
Pakistan	PAK	Eastern Mediterranean	46.16	—	—	—	Available	—	1,455	67.53	1.16 (2019)	Excluded
Palau	PLW	Western Pacific	51.93	—	—	—	Available	—	13,267	—	3.93 (2021)	Excluded
Palestine	PSE	—	40.72	—	—	—	—	—	3,679	—	—	Excluded
Panama	PAN	Americas	41.87	Yes	Yes	Yes	Unavailable	Yes/Unavailable	15,510	54.41	2.88 (2021)	Included
Papua New Guinea	PNG	Western Pacific	48.34	Yes	Yes	Yes	Available	Yes/Available	2,608	26.47	0.05 (2021)	Included
Paraguay	PRY	Americas	49.47	Yes	Yes	Yes	Available	Yes/Available	5,975	40.71	12.84 (2021)	Included
Peru	PER	Americas	43.76	Yes	Yes	Yes	Available	Yes/Available	6,829	48.58	2.18 (2021)	Included
Philippines	PHL	Western Pacific	51.58	No	No	No	Unavailable	No/Unavailable	3,484	55.62	2.51 (2021)	Included
Poland	POL	Europe	55.81	Yes	Yes	Yes	Available	Yes/Available	18,636	125.95	9.17 (2021)	Included
Portugal	PRT	Europe	38.61	No	No	No	Available	No/Available	24,711	49.75	11.70 (2021)	Included
Puerto Rico	PRI	—	47.37	—	—	—	—	—	32,619	—	—	Excluded
Qatar	QAT	Eastern Mediterranean	52.81	—	—	—	Available	—	71,752	63.67	6.53 (2019)	Excluded
Republic of Korea	KOR	Western Pacific	54.48	Yes	Yes	No	—	—	37,518	76.44	5.30 (2021)	Excluded
Republic of Moldova	MDA	Europe	58.97	No	No	Yes	Unavailable	No/Unavailable	5,275	96.38	5.09 (2021)	Included
Romania	ROU	Europe	64.23	No	Yes	Yes	Unavailable	No/Unavailable	14,908	73.64	10.51 (2021)	Included

Country	ISO3	WHO region	Caries %	Advanced	Essential	Preventive	Restorative	Profile	GDP 2021	Sugar g/day	Dentists/10k (year)	Primary
Russian Federation	RUS	Europe	57.70	—	—	—	Available	—	12,425	91.62	2.79 (2020)	Excluded
Rwanda	RWA	Africa	35.91	Yes	Yes	Yes	Available	Yes/Available	843	32.93	0.21 (2021)	Included
Saint Kitts and Nevis	KNA	Americas	49.02	—	—	—	Available	—	18,361	105.42	—	Excluded
Saint Lucia	LCA	Americas	47.27	—	—	—	Available	—	10,481	78.68	—	Excluded
Saint Vincent and the Grenadines	VCT	Americas	47.01	—	—	—	Available	—	8,641	72.79	1.85 (2021)	Excluded
Samoa	WSM	Western Pacific	51.60	—	—	—	Available	—	4,022	79.37	0.90 (2020)	Excluded
San Marino	SMR	Europe	40.97	No	Yes	Yes	Unavailable	No/Unavailable	54,169	—	7.33 (2022)	Excluded
Sao Tome and Principe	STP	Africa	35.36	—	—	—	Available	—	2,363	44.88	0.27 (2022)	Excluded
Saudi Arabia	SAU	Eastern Mediterranean	49.30	—	—	—	Available	—	31,921	91.48	7.26 (2021)	Excluded
Senegal	SEN	Africa	39.50	No	Yes	Yes	Available	No/Available	1,598	44.55	0.10 (2021)	Included
Serbia	SRB	Europe	46.35	No	Yes	Yes	—	—	9,681	34.25	2.45 (2021)	Excluded
Seychelles	SYC	Africa	51.22	—	—	—	Available	—	14,983	63.26	3.51 (2022)	Excluded
Sierra Leone	SLE	Africa	36.20	Yes	Yes	Yes	Unavailable	Yes/Unavailable	885	13.21	0.02 (2020)	Included
Singapore	SGP	Western Pacific	46.87	Yes	Yes	Yes	Available	Yes/Available	80,885	—	4.32 (2021)	Excluded
Slovakia	SVK	Europe	56.28	Yes	Yes	Yes	—	—	22,123	50.33	5.28 (2021)	Excluded
Slovenia	SVN	Europe	56.24	—	—	—	Available	—	29,193	59.32	7.46 (2021)	Excluded
Solomon Islands	SLB	Western Pacific	50.42	Yes	Yes	Yes	Available	Yes/Available	2,043	37.51	0.69 (2019)	Included
Somalia	SOM	Eastern Mediterranean	35.49	No	No	Yes	Unavailable	No/Unavailable	549	—	—	Excluded
South Africa	ZAF	Africa	40.96	Yes	Yes	Yes	Available	Yes/Available	6,829	74.41	1.07 (2021)	Included
South Sudan	SSD	Africa	42.01	No	Yes	Yes	Unavailable	No/Unavailable	—	—	0.03 (2020)	Excluded
Spain	ESP	Europe	38.36	Yes	Yes	Yes	Unavailable	Yes/Unavailable	30,799	80.03	8.47 (2021)	Included
Sri Lanka	LKA	South-East Asia	55.86	Yes	Yes	Yes	Available	Yes/Available	3,997	84.79	1.01 (2021)	Included
Sudan	SDN	Eastern Mediterranean	39.66	—	—	—	Unavailable	—	712	—	2.50 (2020)	Excluded
Suriname	SUR	Americas	50.63	—	—	—	Available	—	5,030	104.25	0.70 (2021)	Excluded
Sweden	SWE	Europe	44.62	Yes	Yes	Yes	Available	Yes/Available	60,648	89.23	7.74 (2021)	Included
Switzerland	CHE	Europe	22.53	No	No	No	Available	No/Available	96,583	103.12	4.06 (2019)	Included
Syrian Arab Republic	SYR	Eastern Mediterranean	42.63	Yes	Yes	Yes	Available	Yes/Available	689	40.19	10.22 (2021)	Included
Taiwan	TWN	—	58.14	—	—	—	—	—	—	68.03	—	Excluded
Tajikistan	TJK	Europe	53.94	No	Yes	Yes	Unavailable	No/Unavailable	897	51.12	1.65 (2021)	Included
Thailand	THA	South-East Asia	54.38	Yes	Yes	Yes	Available	Yes/Available	7,055	111.48	1.13 (2021)	Included

Country	ISO3	WHO region	Caries %	Advanced	Essential	Preventive	Restorative	Profile	GDP 2021	Sugar g/day	Dentists/10k (year)	Primary
Timor-Leste	TLS	South-East Asia	56.65	Yes	Yes	Yes	Available	Yes/Available	2,685	31.45	0.02 (2020)	Included
Togo	TGO	Africa	32.05	—	—	—	Unavailable	—	1,076	—	0.02 (2021)	Excluded
Tokelau	TKL	—	50.08	—	—	—	—	—	—	—	—	Excluded
Tonga	TON	Western Pacific	51.58	—	—	—	Available	—	4,923	41.48	1.42 (2020)	Excluded
Trinidad and Tobago	TTO	Americas	41.34	No	Yes	Yes	Unavailable	No/Unavailable	17,713	105.37	2.99 (2019)	Included
Tunisia	TUN	Eastern Mediterranean	46.94	Yes	Yes	Yes	Available	Yes/Available	3,907	90.55	3.68 (2021)	Included
Turkmenistan	TKM	Europe	55.67	—	—	—	Available	—	3,713	46.00	1.24 (2021)	Excluded
Tuvalu	TUV	Western Pacific	51.49	Yes	Yes	Yes	Available	Yes/Available	6,043	135.95	0.96 (2020)	Included
Türkiye	TUR	Europe	45.71	—	—	—	Available	—	9,982	72.74	4.60 (2021)	Excluded
Uganda	UGA	Africa	41.21	—	—	—	Unavailable	—	883	31.97	0.06 (2020)	Excluded
Ukraine	UKR	Europe	57.09	Yes	Yes	No	—	—	4,776	97.40	—	Excluded
United Arab Emirates	ARE	Eastern Mediterranean	52.36	Yes	Yes	Yes	Available	Yes/Available	44,118	32.38	7.64 (2021)	Included
United Kingdom	GBR	Europe	33.01	Yes	Yes	Yes	Available	Yes/Available	47,696	76.55	5.12 (2021)	Included
United Republic of Tanzania	TZA	Africa	32.27	No	No	No	Available	No/Available	1,160	28.41	0.12 (2022)	Included
United States Virgin Islands	VIR	—	49.07	—	—	—	—	—	42,571	—	—	Excluded
United States of America	USA	Americas	44.32	No	No	No	—	—	71,441	91.21	5.94 (2021)	Excluded
Uruguay	URY	Americas	51.79	Yes	Yes	Yes	Available	Yes/Available	17,882	100.96	2.26 (2021)	Included
Uzbekistan	UZB	Europe	54.31	—	—	—	Available	—	2,370	51.92	1.42 (2021)	Excluded
Vanuatu	VUT	Western Pacific	50.03	—	—	—	Unavailable	—	3,576	38.58	0.31 (2019)	Excluded
Venezuela (Bolivarian Republic of)	VEN	Americas	39.82	—	—	—	Available	—	2,005	81.75	—	Excluded
Vietnam	VNM	Western Pacific	49.95	—	—	—	—	—	3,704	69.78	—	Excluded
Yemen	YEM	Eastern Mediterranean	41.96	—	—	—	—	—	—	57.73	0.17 (2021)	Excluded
Zambia	ZMB	Africa	42.27	No	Yes	Yes	Unavailable	No/Unavailable	1,127	26.08	0.14 (2021)	Included
Zimbabwe	ZWE	Africa	35.93	—	—	—	Available	—	2,614	42.22	0.30 (2021)	Excluded

The dataset contains one record for each of the 204 IHME national-level outcome locations. Missing policy information was retained as missing and was not recoded as the absence of entitlement. Dentist density was obtained for 2021 when available or from the nearest observation within 2019–2023, with ties resolved in favor of the earlier year.

Supplementary Table S3. Assessment of missingness and analytical-sample selection

Panel A. Comparison of countries included and excluded from the primary complete-case analysis

Characteristic	Included	Excluded	Difference / test	p value
Untreated caries prevalence (%), mean ± SD	45.10 ± 7.60	45.69 ± 7.98	Welch t=-0.54; df=197.63	0.590
GDP per capita, median [IQR]	5,800 [2,515, 15,058]	7,808 [2,366, 30,687] (n=103)	Mann–Whitney U=4170	0.149
Sugar (g/day), mean ± SD	67.35 ± 32.96	67.98 ± 29.48 (n=80)	Welch t=-0.13; df=169.86	0.894
WHO-region distribution	n=92	n=102; 10 unavailable	Permutation χ^2=11.66; Cramér V=.245	**0.038**
Africa	23	24		
Americas	20	15		
Eastern Mediterranean	8	13		
Europe	22	31		
South-East Asia	9	1		
Western Pacific	10	18		

Selection comparisons are descriptive, not confirmatory. The WHO-region p value uses 20,000 label permutations; regional selection differences may limit generalizability.

Panel B. Countries excluded after the 101-country policy–service match because of missing core covariates

Country	ISO3	Joint-indicator category	GDP 2021
Benin	BEN	No/Unavailable	1,319
Burundi	BDI	No/Unavailable	264
Cuba	CUB	Yes/Available	Missing
Eritrea	ERI	No/Unavailable	Missing
Mali	MLI	No/Unavailable	1,027
San Marino	SMR	No/Unavailable	54,169
Singapore	SGP	Yes/Available	80,885
Somalia	SOM	No/Unavailable	549
South Sudan	SSD	No/Unavailable	Missing

No country with complete policy/service and sugar data was subsequently excluded because of GDP or WHO-region missingness; final N therefore remained 92. All nine countries were excluded because sugar-availability data were missing. GDP per capita data were additionally missing for Cuba, Eritrea, and South Sudan.

Supplementary Table S4. Sequential country selection and variable-level missingness.

Part A. Sequential sample selection

Selection stage	Remaining n	Excluded at stage
Initial IHME national-level locations	204	-
Non-missing advanced-curative entitlement data	111	93
Non-missing restorative-service data	101	10
Available sugar data	92	9
Complete GDP and WHO-region data	92	0

Panel A shows sequential sample selection. Missing policy or service data were not classified as absent entitlement or unavailable services.

Part B. Variable-level missingness in the initial 204 locations

Variable	Missing n (%)
Untreated caries prevalence	0 (0.0%)
Advanced curative entitlement	93 (45.6%)
Essential curative entitlement	93 (45.6%)
Preventive entitlement	93 (45.6%)
Basic restorative service availability	28 (13.7%)
GDP per capita	9 (4.4%)
Sugar availability	32 (15.7%)
WHO region	10 (4.9%)

Panel B shows missingness among 204 locations. Missing policy or service data were not classified as absent entitlement or unavailable services.

Supplementary Table S5. Games–Howell post hoc comparisons of crude untreated caries prevalence across joint-indicator categories.

Comparison	Mean difference (pp)	95% simultaneous CI	q	df	*p* value
Yes/Available vs Yes/Unavailable	3.63	-2.23 to 9.48	2.45	20.41	0.334
Yes/Available vs No/Available	7.36	2.05 to 12.67	5.39	25.53	**0.004**
Yes/Available vs No/Unavailable	1.11	-6.94 to 9.16	0.56	15.33	0.978
Yes/Unavailable vs No/Available	3.74	-3.12 to 10.59	2.11	27.16	0.457
Yes/Unavailable vs No/Unavailable	-2.51	-11.47 to 6.44	1.10	21.94	0.863
No/Available vs No/Unavailable	-6.25	-14.95 to 2.45	2.83	20.97	0.219

Groups are defined by advanced-curative entitlement (Yes/No) and restorative service availability (Available/Unavailable). Mean differences represent the first group minus the second. Games–Howell comparisons account for unequal variances and multiple testing; 95% CIs are simultaneous. Results are unadjusted. CI = confidence interval; df = degrees of freedom; pp = percentage points; q = studentized range statistic.

Supplementary Table S6. Geographical and economic distribution of included and excluded locations.

Part A. WHO-region distribution by sequential exclusion reason

WHO region	Missing entitlement data (n=93)	Missing restorative-service data (n=10)	Missing sugar data (n=9)	Included (n=92)
Africa	17	2	5	23
Americas	13	1	1	20
Eastern Mediterranean	12	0	1	8
Europe	24	6	1	22
South-East Asia	1	0	0	9
Western Pacific	16	1	1	10
WHO region not recorded	10	0	0	0
Total	93	10	9	92

Part B. Observed GDP-per-capita quartiles by sequential exclusion reason

GDP quartile	Missing advanced entitlement	Missing restorative availability	Missing sugar	Included
Q1 (lowest)	20	3	4	22
Q2	19	1	0	29

Q3	21	1	0	26
Q4 (highest)	27	5	2	15
GDP not recorded	6	0	3	0
Total	93	10	9	92

Exclusion categories are mutually exclusive and reflect the first missing variable. GDP quartiles in Panel B are based on 195 locations with observed GDP and do not represent World Bank income groups.

Supplementary Table S7. Prespecified sensitivity and robustness analyses for the primary entitlement-by-availability interaction

**Sensitivity analysis**	**N**	**Interaction estimate**	**95% CI**	***p* value**
Primary HC3 OLS	92	8.997	1.706 to 16.289	0.016
+ dentist density (nearest 2019-2023)	88	8.596	1.148 to 16.044	0.024
+ dentist density (exact 2021 only)	65	9.169	0.162 to 18.175	0.046
Influential-country exclusion (Cook's D > 4/N)	85	7.260	1.459 to 13.060	0.015
Essential Curative × Restorative alternative	92	8.296	0.237 to 16.355	0.044
Population-weighted WLS	92	6.114	-1.011 to 13.240	0.092
Beta regression (HC3 covariance)	92	0.372	0.109 to 0.634	**0.005**

OLS and WLS estimates are expressed in percentage points; beta-regression estimates are on the logit-mean scale. Inference uses HC3-robust covariance. CI = confidence interval; OLS = ordinary least squares; WLS = weighted least squares.

Supplementary Table S8. Country-level influence diagnostics for observations exceeding prespecified screening thresholds in the primary regression model

**Country**	**Cook's D**	**Leverage**	**Externally studentized residual**	**Flag**
Bhutan	0.0533	0.1686	−1.7214	Cook's D
Chile	0.0713	0.1568	2.0967	Cook's D
Germany	0.0631	0.0683	−3.2548	Cook's D, residual
Romania	0.0917	0.1201	2.8334	Cook's D
Switzerland	0.1135	0.1206	−3.1837	Cook's D, residual
Syrian Arab Republic	0.0599	0.2167	−1.5571	Cook's D
United Arab Emirates	0.0807	0.2323	1.7335	Cook's D

China	0.0090	0.2401	0.5570	Leverage
Tajikistan	0.0005	0.2434	0.1293	Leverage

Screening thresholds were Cook's D > 4/N, leverage > 2k/N (k = 11), and |externally studentized residual| > 3 (N = 92). Only seven countries exceeding the Cook's D threshold were excluded in sensitivity analysis.

Supplementary Table S9. Secondary entitlement models for untreated deciduous caries prevalence

Model	Exposure	N	Adjusted β (pp)	HC3 SE	95% CI	*p* value
Secondary Model 1	Routine/preventive entitlement: Yes vs No	101	2.33	1.84	-1.34 to 5.99	0.211
Secondary Model 2	Essential curative entitlement: Yes vs No	101	3.15	1.80	-0.44 to 6.73	0.085

Each model adjusts for ln(GDP), sugar per 10 g/day, and WHO region using HC3 robust SE. Entitlement indicators were modeled separately to limit redundancy and multicollinearity.

Supplementary Table S10. Temporal interpretation of study indicators.

Indicator	Temporal interpretation
Untreated caries prevalence, ages 1–9 years	Modeled prevalence estimate for 2021; disease may have developed during preceding years
Advanced, essential, and preventive oral-health entitlement	Reported benefit-package inclusion; dates of policy introduction and duration of implementation unavailable
Basic restorative service availability	Reported national service availability; historical availability and duration of service provision unknown
Sugar availability	Contemporaneous national food-supply indicator; does not represent children's cumulative dietary exposure
GDP per capita	Contemporaneous macroeconomic indicator; does not capture historical economic conditions
Dentist density	Some observations originate from different years; used in sensitivity analyses rather than the primary adjustment set

Except for dentist density (2021 or nearest observation within 2019–2023), all indicators refer to 2021.

Supplementary Figures

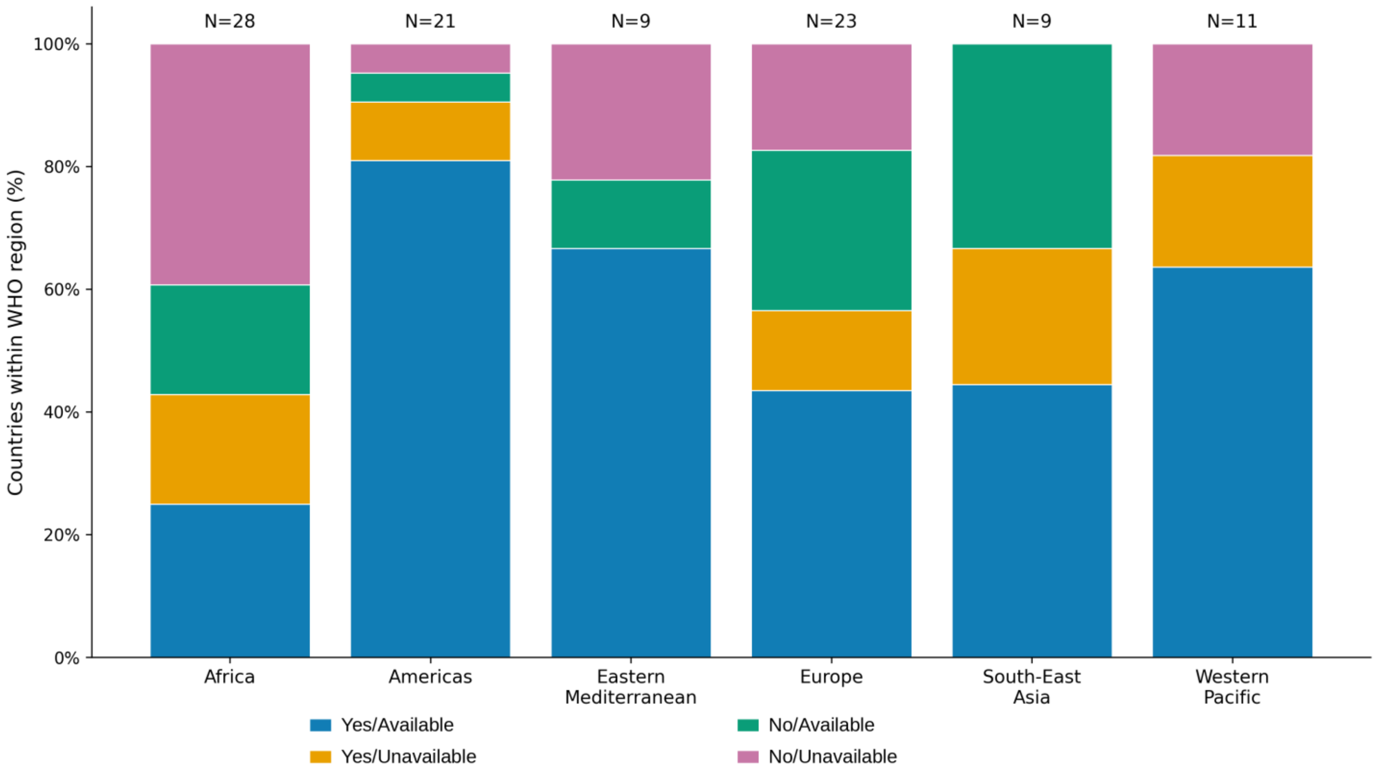

Supplementary Figure S1. Distribution of joint-indicator categories across WHO regions among 101 countries with observed entitlement and restorative-service data.

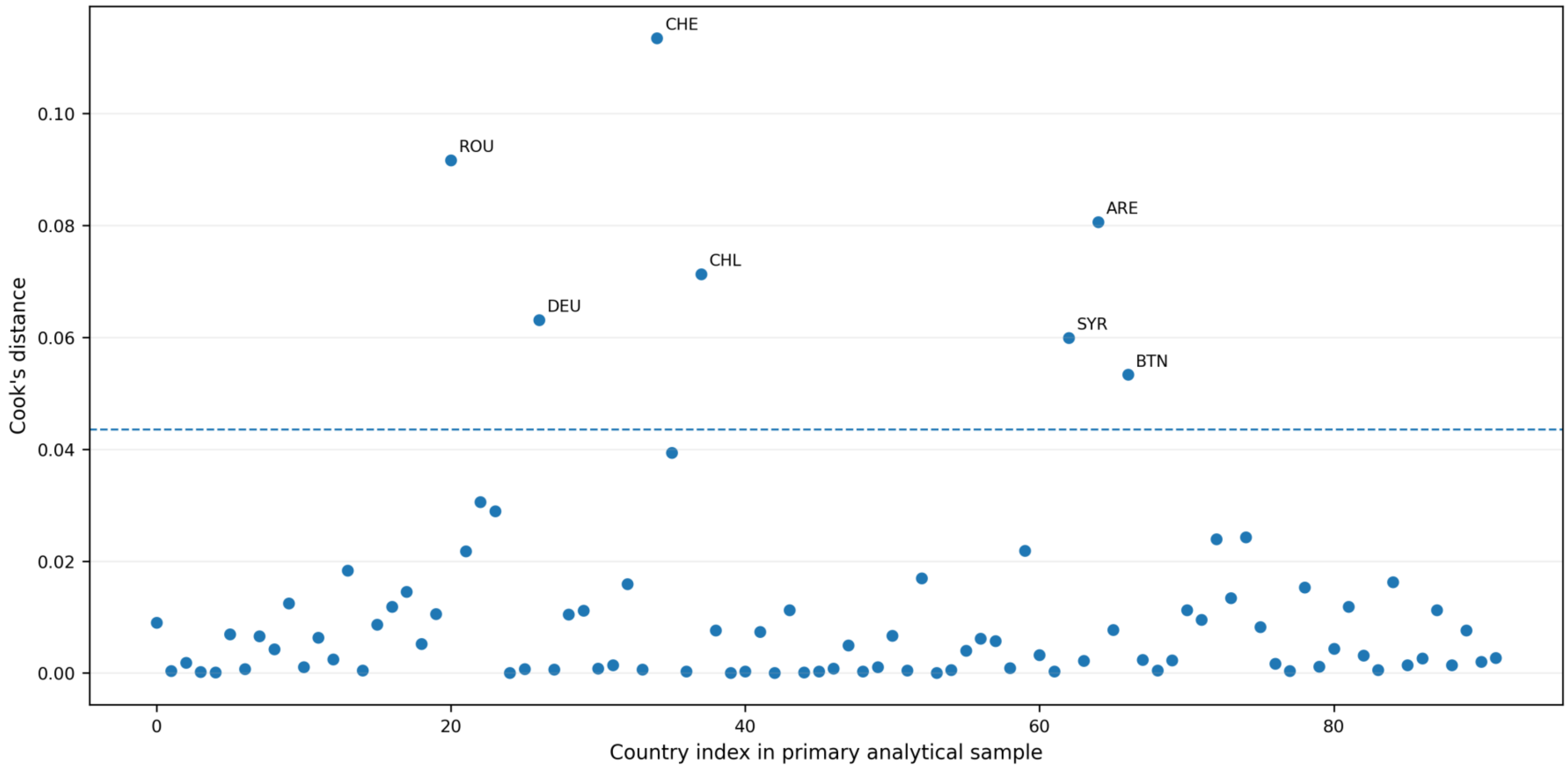

Supplementary Figure S2A. Cook's distance for the primary regression model. Dashed line = 4/N. Seven countries exceeded this screening threshold (ROU, DEU, CHE, CHL, SYR, ARE, BTN); they were retained in the primary model and removed only in sensitivity analysis.

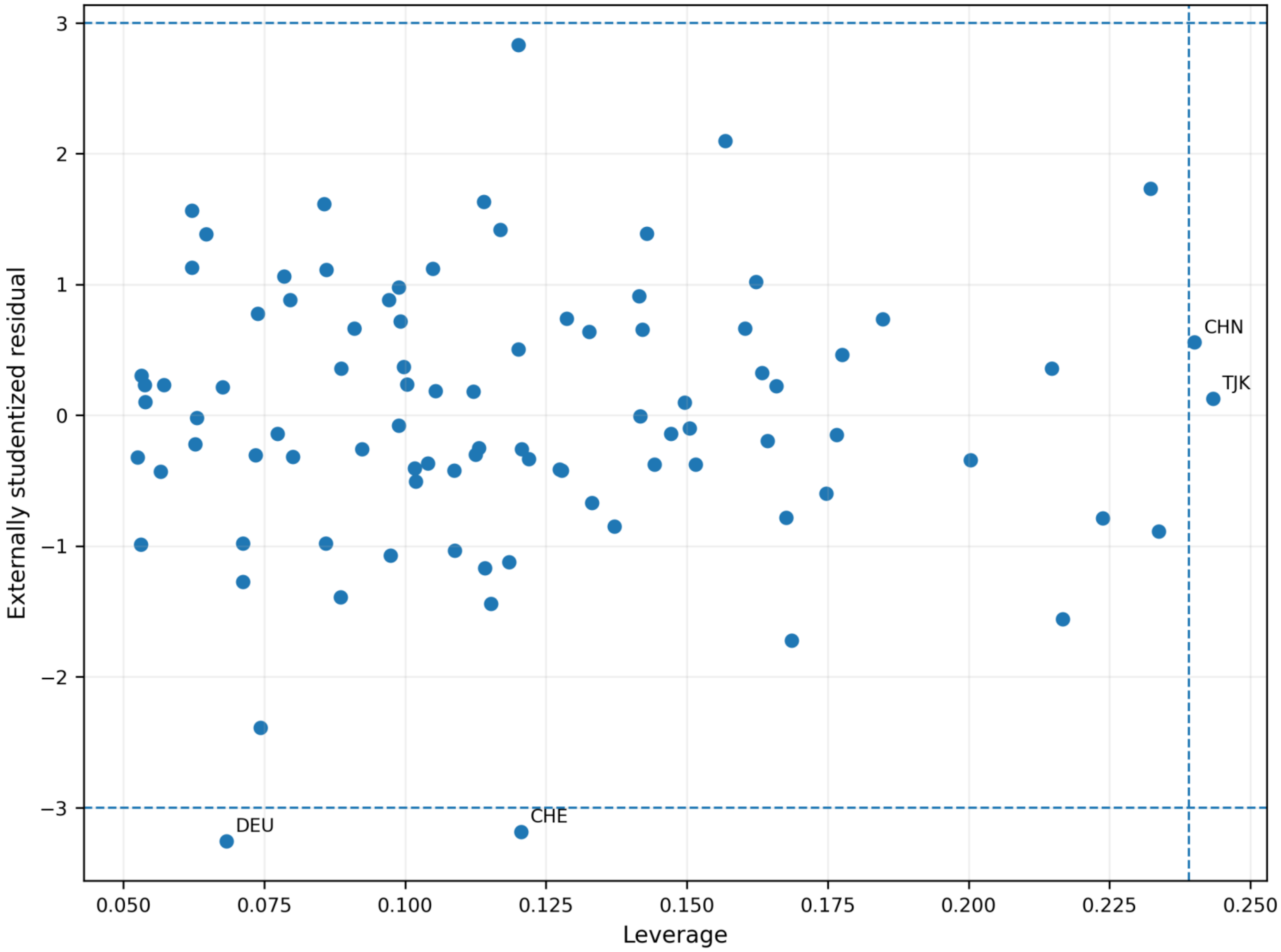

Supplementary Figure S2B. Leverage and externally studentized residual diagnostics. Dashed lines denote leverage >2p/N and |externally studentized residual|>3. China and Tajikistan exceeded the leverage screen; Germany and Switzerland exceeded the studentized-residual screen.
